# One-Month Global Longitudinal Strain Identifies Patients Who Will Develop Pacing-Induced Left Ventricular Dysfunction over Time: The Pacing and Ventricular Dysfunction (PAVD) Study

**DOI:** 10.1371/journal.pone.0162072

**Published:** 2017-01-17

**Authors:** Fozia Zahir Ahmed, Manish Motwani, Colin Cunnington, Chun Shing Kwok, Catherine Fullwood, Delvac Oceandy, Alan Fitchet, Grahame Kevin Goode, Matthew Luckie, Amir Masood Zaidi, Rajdeep Khattar, Mamas Andreas Mamas

**Affiliations:** 1 Cardiovascular Institute, Faculty of Medical and Human Sciences, University of Manchester, Manchester, United Kingdom; 2 Department of Cardiology, Manchester Heart Centre, Manchester Royal Infirmary, Manchester, United Kingdom; 3 Cardiovascular Research Group, Institutes of Science and Technology in Medicine and Primary Care, University of Keele, Keele, United Kingdom; 4 University Hospital North Midlands, Stoke-on-Trent, United Kingdom; 5 Manchester Biomedical Research Centre, Central Manchester University Hospitals NHS Foundation Trust, Manchester Academic Health Sciences Centre, Manchester, United Kingdom; 6 Department of Biostatistics, Institute of Population Health, University of Manchester, Manchester, United Kingdom; 7 Department of Cardiology, Lancashire Cardiac Centre, Blackpool, United Kingdom; 8 Department of Cardiology, Salford Royal Foundation Trust, Stott Lane, Salford, United Kingdom; 9 Department of Cardiology, Royal Brompton Hospital and Cardiovascular Biomedical Research Unit, Imperial College, London, United Kingdom; University of Milano, ITALY

## Abstract

**Background:**

Predicting which individuals will have a decline in left ventricular (LV) function after pacemaker implantation remains an important challenge. We investigated whether LV global longitudinal strain (GLS), measured by 2D speckle tracking strain echocardiography, can identify patients at risk of pacing-induced left ventricular dysfunction (PIVD) or pacing-induced cardiomyopathy (PICMP).

**Methods:**

Fifty-five patients with atrioventricular block and preserved LV function underwent dual-chamber pacemaker implantation and were followed with serial transthoracic echocardiography for 12 months for the development of PIVD (defined as a reduction in LV ejection fraction (LVEF) ≥5 percentage points at 12 months) or PICMP (reduction in LVEF to <45%).

**Results:**

At 12 months, 15 (27%) patients developed PIVD; of these, 4 patients developed PICMP. At one month, GLS was significantly lower in the 15 patients who subsequently developed PIVD, compared to those who did not (n = 40) (GLS -12.6 vs. -16.4 respectively; p = 0.022). When patients with PICMP were excluded, one month GLS was significantly reduced compared to baseline whereas LVEF was not. One-month GLS had high predictive accuracy for determining subsequent development of PIVD or PICMP (AUC = 0.80, optimal GLS threshold: <−14.5, sensitivity 82%, specificity 75%); and particularly PICMP (AUC = 0.86, optimal GLS threshold: <−13.5, sensitivity 100%, specificity 71%).

**Conclusions:**

GLS is a novel predictor of decline in LV systolic function following pacemaker implantation, with the potential to identify patients at risk of PIVD before measurable changes in LVEF are apparent. GLS measured one month after implantation has high predictive accuracy for identifying patients who later develop PIVD or PICMP.

## Introduction

Right ventricular (RV) pacing is associated with a reduction in left ventricular (LV) systolic function, [1] thought to be mediated by pacing-induced ventricular dyssynchrony. [2, 3] The prevalence of heart failure after RV pacing is reported to range from 3–31%, the variation largely depending on the definition and methods used to define heart failure as well as the population examined. [4, 5, 6, 7, 8, 9] Moreover, randomized trials in RV paced individuals have shown a near 3-fold increased risk of hospitalization for heart failure when the cumulative percentage of ventricular pacing (Cum%VP) exceeds 40%. [5, 10] The term pacing-induced cardiomyopathy (PICMP) has been used to describe clinically significant left ventricular systolic dysfunction (ejection fraction <45%) attributable to RV pacing, occurring in the absence of other causes of cardiomyopathy. [4, 7] However, lesser degrees of pacing-induced LV dysfunction (PIVD) have also been observed in up to two-thirds of patients with normal baseline LV function. [3, 11, 12] Despite this, clinical guidelines do not currently recommend routine cardiac imaging after pacemaker implantation, and PICMP/PIVD may therefore go undetected until the onset of heart failure symptoms. [8]

In view of this consideration, a *priori* identification of patients at risk of developing heart failure after RV pacing would be of considerable clinical value as these patients may benefit from heightened clinical surveillance and possible upgrade to biventricular pacing, which has been shown to reverse PIVD and LV remodeling. [13, 14, 15] A non-invasive test able to identify such individuals is, therefore, highly desirable. Two-dimensional (2D) speckle tracking strain echocardiography (STE) has been shown to detect early signs of LV systolic dysfunction in a range of cardiomyopathies before a measurable reduction in LVEF. [16, 17, 18, 19, 20]

### Objectives

We investigated whether STE can be used to predict the development of PIVD and PICMP after pacemaker implantation.

## Methods

### Subjects

The Pacing And Ventricular Dysfunction (PAVD) Study is an investigator-initiated prospective observational cohort study designed to investigate predictors of PIVD. Sixty subjects with high-grade atrioventricular block scheduled to undergo dual chamber pacemaker implantation were recruited from three UK centres. Eligible patients were aged 18 years or over, had preserved LVEF (≥55%) and second-degree or third-degree atrioventricular block. All patients were required to provide written informed consent for inclusion. Exclusion criteria were: pregnancy, myocardial infarction or coronary revascularization within prior 3 months, atrial fibrillation (AF), haemodynamically significant valvular heart disease (≥moderate in severity), structural heart abnormality including LV dilatation (according to British Society of Echocardiography reference ranges), LVEF <55%, New York Heart Association (NYHA) functional class III or IV, significant respiratory disease, history of carcinoma within 5 years, autoimmune disorders, rheumatoid arthritis or treatment with disease modifying drugs including mineralocorticoid receptor antagonists.

### Study protocol

The Central Manchester research ethics committee and institutional review board reviewed and approved the current clinical study in 2012. All clinical investigations were conducted according to the principles expressed in the Declaration of Helsinki. Written informed consent was obtained from all participants. Baseline evaluation consisted of (i) assessment of NYHA functional class and age-adjusted Charlson score [21] (ii) 12-lead electrocardiogram (ECG) (iii) standard transthoracic echocardiography. The protocol required that patients undergo standardized implantation of a dual chamber permanent pacemaker with positioning of the ventricular lead at the RV apex. Fluoroscopy and x-ray were used to record the anatomical location of the RV lead in each case. Pacemakers were programmed to DDDR mode with the manufacturer’s algorithms to minimize ventricular pacing enabled. Measurements (i)-(iii) were repeated at 1 and 12 months after pacemaker implantation, as well as measurement of the cumulative percentage of ventricular pacing (Cum%VP) from stored pacemaker diagnostics. At follow-up, where the patient was not paced at the time of undertaking pacemaker interrogation, pacing at 60 pulses per minute was enabled for the duration of the echocardiographic examination.

### Echocardiography

#### Image acquisition

Standard two-dimensional echocardiography was performed before and after pacemaker implantation (baseline, 1 and 12 months) using an iE-33 ultrasound system with a 1–5MHz phased-array probe (Philips Medical System, Andover, USA). Examinations were performed in the left lateral decubitus position by an experienced operator. Acquisitions from standard parasternal long-axis, apical and LV short axis views were obtained at end-expiration with sector width and depth optimized to allow for complete myocardial visualization while maximizing frame rate (60–110 frames per second). Cine loops were acquired for 3 consecutive cycles. Two-dimensional LVEF were measured using Simpson’s biplane method. [22] PIVD was defined as an absolute decline in LVEF by ≥5 percentage points. PICMP was defined as a reduction in LVEF to <45%. A single experienced operator who was blinded to the pacing interrogation results performed all echocardiographic analyses.

#### Speckle-tracking strain analysis

Strain was evaluated off-line from digitally stored images using Qlab 9 (cardiac motion quantification (CMQ); Phillips Medical Systems) software package. Longitudinal strain for individual myocardial segments was measured from the apical four-chamber, two-chamber and long axis views (16 segment AHA/ASE model). [23] In end-diastole, automated border tracking was enabled, before manual adjustment using a point and click approach to ensure that the endocardial and epicardial borders were included in the region of interest. In cases of poor tracking, fine-tuning was performed manually after cineloop playback and tracing was repeated and adjusted until tracking was considered optimal by visual analysis. Individual segments that returned positive strain values, and those with persistently poor tracking despite manual optimisation, were excluded from analysis. Peak strain for the segment was defined as the peak negative value on the time strain curve for the entire cardiac cycle. Peak regional longitudinal strain was measured in 16 myocardial regions and a weighted mean was used to derive global longitudinal strain (GLS).

Longitudinal LV dyssynchrony was evaluated using the standard deviation of time to peak strain (TPS-SD), as previously described. [24] Briefly, time to peak strain was measured from the interval from onset of the Q wave to peak negative strain throughout the cardiac cycle. Time-strain curves for the 12 basal and mid LV segments were generated and the TPS-SD for these segments was calculated. [24] Dyssynchrony was defined as TPS-SD >60ms.

### Reproducibility of data

Echocardiographic images for ten patients were independently analyzed twice, at intervals of greater than 2 weeks, by two investigators (FZA and ML). Both operators were blinded to the results of the first measurement and from each other. Intraobserver variability for GLS measurements was small (mean arithmetic difference in GLS 0.41, (3.3%)). Intraobserver variability for LVEF was 4.2%. Interobserver variability for GLS and LVEF was 5.4% and 5.1% respectively.

### Statistical analysis

Continuous data are presented as mean ± SD. Categorical data are presented as frequencies and percentages. Normally distributed variables were compared using an unpaired t-test with Welch’s correction. Categorical data were compared using the Fisher’s exact test. Non-normally distributed data were compared using the Mann-Whitney U-test or Kruskall-Wallis test as appropriate. Statistical analyses were performed using GraphPad Prism (version 6.0e for Mac, GraphPad Software, San Diego, California, USA) and Stata (StataCorp. 2015. Stata Statistical Software: Release 14. College Station, TX: StataCorp LP. Receiver operating characteristic (ROC) analysis was performed to determine the accuracy of one month GLS to predict (i) PIVD including PICMP and (ii) specifically PICMP. The optimal GLS thresholds from the ROC curve were determined using the maximum Youden index. [25] Multivariate logistic regression analysis was performed to identify predictors of PIVD. (S1 File).

## Results

### Study population

**Fig 1:**
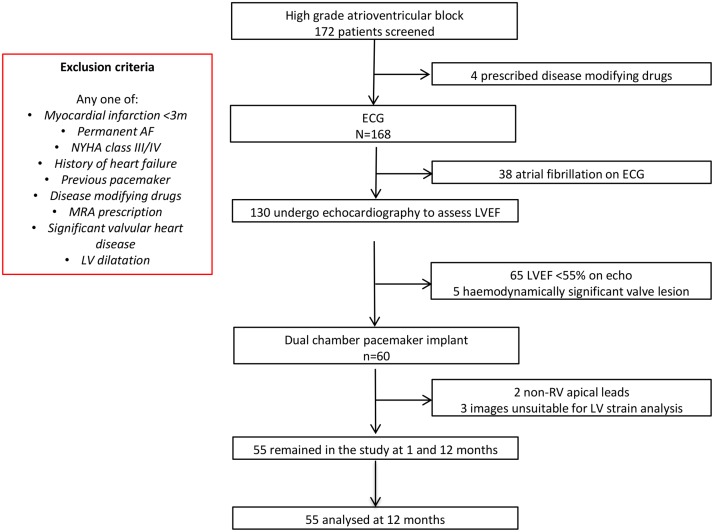
Patient distribution through the study at 12 months. Algorithm showing total number of cases considered for recruitment and the reasons for exclusion, leading to selection of the final 55 patients.

Between October 2012 and May 2014, 172 patients were screened for the study. Sixty patients with second or third-degree atrioventricular block and preserved LV systolic function fulfilled criteria for inclusion. After pacemaker implantation, five patients were excluded due to suboptimal echocardiographic images for GLS measurement (n = 3) or non-apical RV pacing leads (n = 2). Therefore, the study population consisted of 55 patients (72.2 ±14.4 years; 35 (64%) men) of whom 2 were undergoing temporary transvenous pacing prior to permanent pacemaker implantation. Baseline clinical characteristics for the study participants are given in Table 1. Fig 1 outlines the patient distribution for the study.

**Table 1:** Clinical characteristics between patients with low and high burdens of ventricular pacing.

Variable	All (n = 55)	Decline in LVEF (n = 15)	No decline in LVEF (n = 40)	p
Age	72.7 + 13.5	72.4 + 13.4	72.8 + 13.8	0.937
Male (%)	35 (63)	12	23	0.208
Baseline LVEF (%)	61.1 + 5.4	60.7 + 6.2	61.3 + 5.1	0.708
Diabetes (%)	9 (16)	2	7	0.633
Hypertension (%)	23 (42)	3	20	0.066
IHD (%)	9 (16)	3	6	0.692
Paroxysmal AF (%)	6 (11)	3	3	0.329
Age adjusted Charlson Score	3.0 + 1.6	2.7 + 1.7	3.1 + 1.6	0.566
NYHA class I (%)	34 (62)	12	22	0.378
NYHA class II (%)	21 (38)	3	18	0.123
Betablocker (%)	5 (9)	2	3	0.606
Ace-inhibitor	7 (13)	3	4	0.376
ARB (%)	6 (11)	0	6	0.319
Calcium channel blocker (%)	5 (9)	1	4	1.000
Diuretics (%)	2 (4)	1	2	0.474
HR pre pacemaker	55.6 + 10.3	51.6 + 14.6	56.7 + 8.9	0.404
TPW pre ppm (%)	2 (4)	1	1	0.474
Second degree AV block	45 (82)	10	35	0.115
CHB (%)	10 (18)	5	5	0.115
Pre-pacing QRS duration	104.4 + 26.6	107.5 + 28.4	103.2 +26.5	0.717
Post-pacing QRS duration	137.3 +46.3	152.7 + 53.0	125.0 + 37.8	0.163
Baseline TPS SD	40.0 + 39.0	45.7 + 30.2	37.6 + 43.1	0.666
TPS SD > 60ms at baseline (%)	4 (7)	2	2	0.298
Post-pacing TPS SD	51.6 + 46.3	64.0 + 52	48.4 + 44.7	0.382
TPS SD >60ms post pacing	14 (25)	5	9	0.493
Mean Cum%AP at 12m	35.0 + 33.7	51.6 + 35.7	30.3 + 31.6	0.119
Mean Cum%VP at 12m	53.5 + 45.0	86.8 + 33.0	43.7 +43.2	0.005

### Incidence of ventricular dysfunction

PIVD occurred in 8/55 (15%) patients at 1 month, but there were no cases of PICMP at this timepoint. At 12 months, PIVD was observed in 15/55 (27%) patients, including 4 patients who reached criteria for diagnosis of PICMP (LVEF <45%). In these 15 patients, one-month LVEF was significantly reduced compared to baseline [LVEF 52.5 ± 6.5 vs. 60.7 ± 6.2 respectively, p<0.05] (Table 2). However, when the 4 cases of PICMP were excluded from the analysis to leave just cases of PIVD (n = 11), 1 month LVEF was not significantly reduced compared to baseline [LVEF 55.6 ±6.6 vs. 62.3 ± 6.8 respectively, p = ns] (Table 3).

**Table 2:** Differences in LVEF and GLS values between patients with and without pacing-induced LV dysfunction.

	Decline in LVEF PIVD and PICMP cases (n = 15)	No decline in LVEF (n = 40)	p
**LVEF**			
Baseline	60.7 + 6.2	61.3 + 5.1	0.780
1 month	52.5 + 6.5*	60.4 + 4.5	0.002
12 months	46.7 + 8.9*	58.7 4.5	0.010
**Global longitudinal strain**			
Baseline	-16.3 + 0.5	-17.5 + 0.6	0.515
1 month	-12.6 + 0.9*	-16.4 + 0.6	0.022
12 months	-11.9 + 2.5*	-15.8 + 3.9	0.008

* denotes significantly reduced compared to baseline measurement (p<0.05).

**Table 3:** Differences in LVEF and GLS in patients with a decline in LVEF (PICMP and PIVD) compared to cases without a decline in LVEF.

	Decline in LVEF		No decline in LVEF	p
	PICMP (n = 4)	PIVD (n = 11)	(n = 40)	
**LVEF**				
Baseline	57.5 + 2.6	62.3 + 6.8	61.3 + 5.1	0.217
1 month	48.3 + 4.2*	55.6 + 6.6	60.4 + 4.5	<0.0001
12 months	41.0 + 4.3*	53.8 + 6.7*	58.7 4.5	<0.0001
**Global longitudinal strain**				
Baseline	-16.0 + 0.8	-16.4 + 0.7	-17.5 + 0.6	0.881
1 month	-11.3 + 2.1*	-13.3 + 1.2*	-16.4 + 0.6	0.005
12 months	-9.8 + 1.7*	-12.8 + 2.6*	-15.8 + 3.9	0.013

* denotes significantly reduced compared to baseline measurement (p<0.05).

### Global longitudinal strain as a predictor of left ventricular dysfunction

Baseline GLS values were not significantly different between patients who developed PIVD at 12 months and those who did not. However, one-month GLS values were significantly lower in the group who went on to develop PIVD at 12 months (excluding PICMP) compared to those who did not [-13.3 ±1.2 vs. -16.4 ±0.6 respectively; p = 0.044] (Table 3).

**Fig 2:**
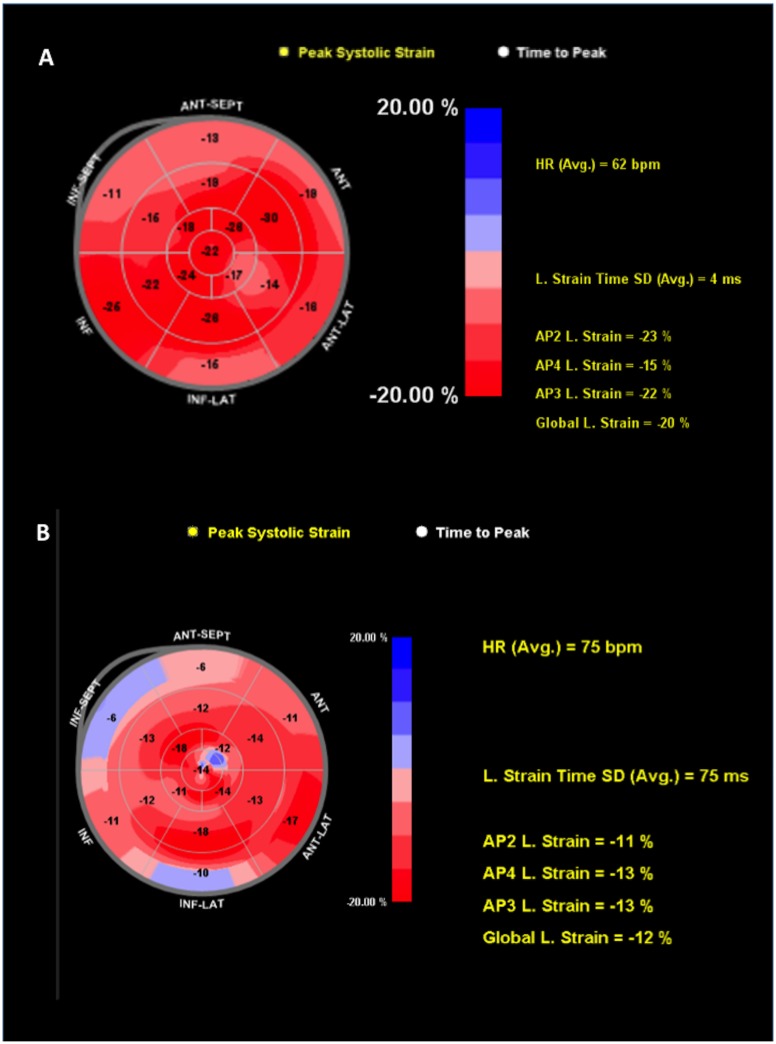
Examples of normal and abnormal polar plot strain maps. (A) Normal polar plot map (GLS -20%). (B) Abnormal polar plot map from a patient who developed PICMP by 12 months. GLS and TPS-SD measure -12% and 75ms respectively, indicating reduced global longitudinal strain and LV dyssynchrony.

**Fig 3:**
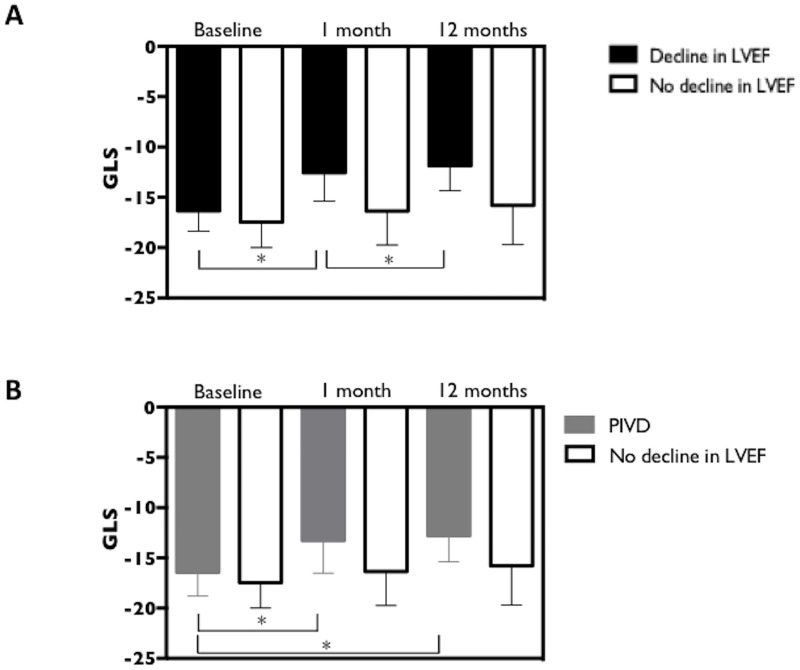
(A) Global longitudinal strain for cases of all cases of PIVD (PICMP included). Global longitudinal strain was significantly lower in patients with a decline in LVEF ≥ 5% compared to cases without (one month GLS -12.6 ± 0.9 vs. -16.4 ±0.6 respectively; p = 0.022). One and 12 month GLS were reduced compared to baseline for cases of with a decline in LVEF at 12 months (PIVD and PICMP), but not for cases without a decline in LVEF (PIVD and PICMP: baseline GLS, -16.3 ±0.5 vs. -12.6 ±0.9 and -11.9 ±2.5; p = 0.012. No decline in LVEF: baseline GLS -17.5 ±0.6 vs. -16.4 ±0.6 and -15.8 ±3.9; p = 0.311). (B) Global longitudinal strain for cases with PIVD (PICMP excluded). One and 12 month GLS were significantly reduced for cases of PIVD compared to baseline (Baseline GLS -16.4 ±0.7 vs -13.3 ±.2 and -12.8 ±2.6 respectively; p = 0.024).

**Fig 4:**
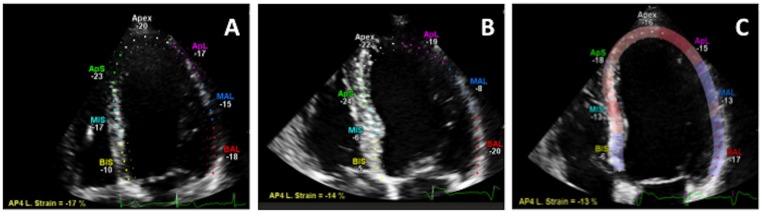
Global longitudinal strain analysis. **(A)** pre pacemaker implant, **(B)** one-month after the initiation of pacing in a patient who developed PIVD and **(C)** 12 months after pacing in a patient who went on to develop PICMP.

Baseline GLS values for those who subsequently developed PICMP (n = 4) were not significantly different from those that did not (Table 3, [-16.0 ±0.8 vs. -16.4 ± 0.7 vs. -17.5 ±0.6 respectively, for cases of PICMP compared to PIVD and cases without PIVD], p = 0.881). At one-month following pacemaker implantation both LVEF and GLS were significantly reduced in these patients (Table 3, [LVEF 48.3 ±4.2, p<0.0001 and GLS -11.3 ±2.1, p = 0.005]) (Figs 2 and 3A). However, in patients that developed PIVD without PICMP (n = 11) only GLS was significantly reduced at one month (Table 3) (Figs 3B and 4).

One-month GLS measurement had a high predictive accuracy for the development of PIVD (including cases of PICMP) at 12 months (area under curve (AUC) = 0.80); the optimal cut-off value of <−14.5 gave a sensitivity of 82% and specificity 75%. The optimal cut-off value for PICMP specifically was <−13.5, with sensitivity 100% and specificity 71%, AUC = 0.86.

Multivariate logistic regression analysis (after adjustment for age, sex, complete heart block, AF, hypertension, ischaemic heart disease, diabetes, NYHA classification and QRS duration >160ms) confirmed that one month GLS <−14.5 was an was an independent predictor of PIVD (OR 24.77 [95% CI:1.78–344.70], p = 0.017). After further adjustment for Cum%VP (greater than 40%), GLS remained an independent predictor of PIVD, (OR 19.09 [95% CI: 1.37–266.34], p = 0.028) (S1 Table).

## Discussion

Although the majority of patients who are RV paced do not develop heart failure related to pacing, LV systolic dysfunction following RV pacing is frequently observed. [7, 8, 9, 11, 12] However, the ability to predict which patients will be affected remains a clinical challenge. The utility of GLS, measured by 2D STE, to identify subclinical LV dysfunction in other conditions has been reported, e.g. following chemotherapy or in patients with known genetic mutations associated with hypertrophic cardiomyopathy but apparently normal or increased LVEF [19, 20, 26, 27, 28]. GLS has also been investigated as a measure of LV dysfunction following pacing. [3, 29, 30, 31] However, this is the first study specifically designed to prospectively examine the temporal relationship between the initiation of RV pacing and serial changes in GLS and LVEF. We hypothesized that GLS may be reduced before significant reductions in LVEF were apparent.

In the present study we aimed to provide a comprehensive analysis of both LVEF and GLS, combining pre-implant data with both short- and medium-term follow-up measurements, in a ‘real world’ study population undergoing RV pacing. Our study is the first to prospectively examine temporal changes in GLS and LVEF, from initiation of RVA pacing through to 12-month follow-up. We observed a significant reduction in both GLS and LVEF at one month following pacemaker implantation in patients exhibiting PICMP (the most severe form of PIVD) at 12 months. More importantly, however, we have shown that GLS measured at one month following pacemaker implantation, but not LVEF, can identify a subgroup of patients who exhibit evidence of PIVD at 12 months. Thus, GLS may represent a clinically useful tool to identify this group of patients who may benefit from heightened echocardiographic surveillance of LV dysfunction following pacemaker implantation. Furthermore, a GLS of <−14.5 at one month had high sensitivity for predicting the development of PIVD (including PICMP) at 12-months, with a value below this threshold being associated with a 19-fold increased risk of developing PIVD. Importantly, this finding was independent of high pacing burden (Cum%VP >40%) in multivariate logistic regression analysis. The wide confidence intervals observed in the multivariate analysis are notable, and in part due to the small sample size. We observed that adjustments made the confidence intervals wider, indicating that the estimates are sensitive to multiple variables (supplementary file), further supporting the need for better powered studies in the future. Although this study identified a one-month GLS threshold, beyond which PIVD was more likely, we did not specifically identify a critical burden of Cum%VP beyond which changes in GLS would be observed, though acknowledge that it would be desirable to explore this in future studies.

Our finding of reduced GLS after RV apical (RVA) pacing is consistent with previous studies. [3, 29, 30, 31] However, previous studies have considered either acute or chronic effects of RVA pacing in isolation; serial assessment of GLS compared to LVEF has not been systematically evaluated. Furthermore, these studies have often been limited by focusing on individual measurements (e.g. GLS or LVEF, rather than evaluating both parameters in the same subjects), single time-point follow-up, or highly selected populations making no allowance for variations in pacing burden (e.g. pacing-dependent patients only, or following AV nodal ablation). [3, 29, 30, 31] For example, Delgado *et al*. examined the acute effects of RVA pacing in patients with preserved LV function and reported a significant decline in both LVEF and GLS, but the long term impact and risk of PIVD progression or PICMP was not described. [31] In a sub-study of the Protection of Left Ventricular Function During Right Ventricular Pacing (Protect-PACE) trial, there was no significant difference in baseline GLS between the RVA pacing group and controls (RV high-septal pacing). [30] Notably, however, GLS was not evaluated prior to pacing, with the baseline measurement being obtained after pacemaker implantation. After 2-years, GLS was reported to be significantly reduced in the RVA group compared to controls, but this study does not yield information regarding the tempo of changes from baseline to 2 years, nor if there was an earlier timepoint for possible therapeutic intervention. [30] Similarly, Ahmed *et al*. evaluated the effects of RV apical pacing on LVEF at 2 years. In a retrospective study, predictors of a decline of LVEF >5% were examined among patients undergoing AV node ablation for atrial fibrillation and pacemaker implantation. GLS performed a median of 4-months after initiation of pacing was significantly reduced in patients who had a decline in LVEF >5% at 2-years compared to those who did not. [3]

The Pacing and Cardiac Enlargement (PACE) study was a prospective study that compared measured LVEF in patients randomized to RVA or biventricular pacing. [4] In RVA patients with PIVD at 1 year, [4] further significant reductions in LVEF were observed when follow-up was extended to 2 years (7% vs. 9.9% reduction in LVEF at 1 and 2 years respectively). [11] Thus, relatively small reductions in LVEF at 12 months, such as those observed in the subjects in our study, may progress to result in more clinically significant reductions in LVEF with extended follow-up, emphasizing the importance of GLS in identifying this patient group at an early stage of the disease process. Moreover, in the present study, a significant decline in both one-month GLS and LVEF was observed in cases that subsequently developed PICMP at 12 months, but a decline in GLS alone at one month in those that developed less severe PIVD. Therefore, we demonstrate the utility of one-month GLS to not only risk stratify patients following pacemaker implantation, but also to predict the magnitude of the ensuing decline in LVEF when the presence of absence of a decline in LVEF of >5% was also considered.

A reduction in LVEF to <50% has important clinical implications. Although the aetiology of LV dysfunction differs and direct comparisons cannot be made, in the Framingham study, LVEF between 40% and 50%, even if asymptomatic, was associated with a nearly four-fold increase in the risk of heart failure and a 1.9-fold increase in the risk of mortality compared to patients with an LVEF >50%. [32] In the current study, by 12 months, 15 patients (80% of whom had a high burden of ventricular pacing) had a significant decline in LVEF ≥ 5 percentage points, of whom 4 had a more severe decline in LVEF to <45% i.e. reaching the threshold for diagnosis of PICMP. Absolute decline in LVEF at 12 months for cases of PICMP (n = 4) was 16.5 percentage points compared to 8.4 percentage points for the remaining less severe PIVD (n = 11) cases (p = 0.029). Though PICMP may be considered a more clinically important situation that requires expedient consideration of upgrade to biventricular pacing, the clinical relevance of asymptomatic “smaller” reductions in LVEF has been widely studied and should not be overlooked as progressive declines in LVEF have been reported with extended follow-up. [3, 4, 11, 32] Although the latter subgroup of patients with PICMP may seem small, the current study postulates that simple screening using echocardiography (inexpensive and widely available) should be considered in order to capture these high-risk patients at an earlier time-point, especially considering the potential long-term health economic burden that is associated with heart failure.

Serial assessment of LVEF has become widely accepted as a research tool for measuring the deleterious effects of RV pacing on LV systolic function. However, the main disadvantage of this practice is that a significant reduction in LVEF may represent the final phenotype of a pathophysiological process. [33] In contrast, abnormal GLS may represent an earlier stage in the disease process before a significant reduction in LVEF occurs, as was observed in cases of PIVD in this study.[19, 20]

## Limitations

Only 35 patients had non-contrast images suitable for three-dimensional (3D) analysis. Therefore, in this study LVEF was calculated using semi-automated non-contrast 2D methods, a practice that more closely reflects real world clinical practice. In this study the cut-off GLS to detect PIVD was calculated to be -14.5 and therefore reported to one decimal place. Although the values of GLS obtained using different vendor software is considered interchangeable, Qlab software itself reports GLS values as a whole integer. This limitation has been considered by previous studies also.

Alternative pacing sites were not examined in the current study. However, as septal pacing is routinely performed in real world practice, it would be desirable for future studies of RV pacing to establish whether one-month GLS significantly differs between RVA and septal pacing sites. Four patients developed PICMP; due to the small sample size, and because all patients who had PICMP had a 1 month GLS <14.5, multivariate logistic regression analysis could not be performed for this cohort. Because of the lower incidence of PICMP compared to lesser degrees of PIVD, such a study would have required significantly more patients and longer follow-up. In addition, as outlined in the discussion, the observation that the confidence interval widens with multiple adjustments indicates that the model is sensitive to multiple adjustments (supplementary file). In view of these considerations, a larger study is needed to validate the feasibility and clinical utility of GLS to predict PIVD.

Finally, although both clinical and animal models have previously explored the temporal relationship between RV pacing, LV dimensions and LVEF, few have expanded this analysis to also take into account GLS. It would be desirable for future clinical studies to examine a more extensive set of echocardiographic variables in order to establish whether those individuals with reduced GLS had subtle abnormalities in LV dimensions and diastolic function at baseline.

## Conclusion

GLS is easily performed in the clinical setting, and when measured at one month following pacemaker implantation shows potential for identifying patients at high risk of subsequent development of PIVD or PICMP, at a time when standard echocardiographic measurements such as LVEF may be unchanged from baseline. Patients with abnormal GLS at one month may benefit from more intensive clinical follow-up and echocardiographic surveillance, with a view to upgrade to biventricular pacing. The utility of GLS in this setting warrants further research.

## Supporting Information

S1 File The minimal dataset used to perform the multivariate analysis is presented herein. (PDF)

S1 Table Effect of global longitudinal strain <14.5 compared to >14.5 on risk of decline in LVEF ≥5% at 12 months according to level of adjustments. (DOCX)
