# Supplementary material for: One-Month Global Longitudinal Strain Identifies Patients Who Will Develop Pacing-Induced Left Ventricular Dysfunction over Time: The Pacing and Ventricular Dysfunction (PAVD) Study
Source: PLoS One. 2017 Jan 17;12(1):e0162072. doi: 10.1371/journal.pone.0162072 (PMC5240943; doi:10.1371/journal.pone.0162072)
Supplement: S1 File — (PDF) [file pone.0162072.s001.pdf]

EPISODE	GROUP(VP>40% = 1, VP<40% = 2)	1MGLS <14.5 (1= YES, 2 = NO)	PIVD at 1 month (decline in LVEF at 1 month >5%)	PIVD (DECLINE IN LVEF >5% AT 12 MONTHS, 1= YES, 0 = NO)	PICMP, LVEF<45% AT 12M (1= YES, 0 = NO)	AGE (1= <70, 2 = >70)	MALE	CHB (1= YES,	AF (1= AF, 2= NO AF)	HYPERTENSION (1= YES, 2= NO)	IHD (1= YES, 2= NO)	DIABETES (1= YES, 2= NO)	NYHA CLASS(1 OR 2)	PACED QRSD >160
101	1	1	0	1	0	1	0	1	1	2	2	2	1	1
102	1	2	0	1	0	1	1	1	2	2	2	2	1	1
103	1	1	0	0	0	1	0	2	2	2	2	2	1	2
104	1	1	0	0	0	0	0	2	2	2	2	2	1	2
105	1	1	0	0	0	1	0	2	2	2	2	2	1	2
106	1	1	0	1	0	0	1	2	1	2	2	2	1	2
107	1	1	0	1	0	1	1	2	2	2	2	2	1	2
108	1	1	1	1	1	1	1	2	2	1	2	2	1	1
109	1	1	1	1	1	1	1	1	2	2	2	2	2	2
110	1	2	0	0	0	0	0	2	2	2	2	2	2	2
112	1	2	0	0	0	1	0	2	2	2	2	2	1	2
113	1	1	0	0	0	0	0	2	1	1	2	2	1	2
115	1	1	1	1	1	0	1	1	2	2	2	2	1	1
116	1	1	0	1	0	1	1	2	2	1	2	2	1	1
117	1	2	0	0	0	0	0	2	2	2	2	2	2	2
121	1	2	0	0	0	0	1	2	2	2	2	2	1	1
122	1	1	0	1	0	0	0	1	2	1	2	1	1	1
125	1	1	0	1	1	0	1	2	2	2	2	2	2	2
126	1	1	1	0	0	1	1	2	2	1	1	2	1	1
130	1	2	0	0	0	0	0	1	2	2	2	2	1	2
134	1	2	0	0	0	0	0	2	2	2	2	2	2	2
135	1	2	0	0	0	1	0	1	2	1	2	1	1	2
137	1	2	0	0	0	1	1	2	2	1	1	2	1	2
138	1	2	0	0	0	0	1	2	2	2	2	2	2	2
146	1	1	0	0	0	1	0	1	2	1	2	1	2	2
151	1	2	1	1	0	0	1	2	2	1	2	2	1	2
153	1	1	0	1	0	1	1	2	2	2	1	1	1	1
154	1	2	0	0	0	1	1	2	2	1	2	1	2	2
159	1	2	0	0	0	0	0	1	2	2	2	2	1	2
111	1	2	0	0	0	1	0	2	2	2	2	2	1	2
114	2	1	0	0	0	0	1	2	2	2	2	2	1	2
118	2	2	0	0	0	0	1	1	2	1	2	2	1	2
119	2	1	0	0	0	1	1	2	2	1	2	2	1	2
120	2	2	0	0	0	0	1	2	2	2	2	2	1	2
123	2	2	0	0	0	1	0	2	1	1	1	2	1	2
124	2	2	1	1	0	0	1	2	2	2	1	1	1	2
127	2	1	0	0	0	0	1	2	2	2	2	2	2	2
128	2	2	0	0	0	0	1	2	2	2	2	2	2	2
129	2	2	0	0	0	0	1	2	2	2	2	2	2	2
131	2	2	0	0	0	0	1	2	2	2	2	2	2	2
132	2	2	0	0	0	0	0	2	2	2	2	2	2	2
133	2	2	0	0	0	0	0	2	2	2	2	2	2	2
136	2	2	0	0	0	0	0	2	2	1	2	2	1	2
139	2	1	0	0	0	1	0	2	2	1	2	2	2	2
140	2	2	0	0	0	1	1	2	2	1	2	1	1	2
141	2	2	0	0	0	0	1	2	2	2	2	2	2	2
142	2	2	0	0	0	0	0	2	2	2	2	2	1	2
143	2	1	0	0	0	1	0	2	1	1	1	2	1	2
144	2	2	0	0	0	0	1	2	2	2	2	2	1	2
145	2	1	1	1	0	0	0	2	2	2	2	2	1	2
147	2	2	0	0	0	1	1	2	2	1	1	2	1	2
148	2	2	0	0	0	1	1	2	2	1	1	1	1	1
149	2	2	0	0	0	1	1	2	2	1	2	1	2	2
150	2	2	0	0	0	1	0	2	2	1	2	2	2	2
152	2	2	1	1	0	1	1	2	1	2	2	2	1	1
TOTALS		22	8	15										
