# Supplementary material for: One-Month Global Longitudinal Strain Identifies Patients Who Will Develop Pacing-Induced Left Ventricular Dysfunction over Time: The Pacing and Ventricular Dysfunction (PAVD) Study
Source: PLoS One. 2017 Jan 17;12(1):e0162072. doi: 10.1371/journal.pone.0162072 (PMC5240943; doi:10.1371/journal.pone.0162072)
Supplement: S1 Table — (DOCX) [file pone.0162072.s002.docx]

| **Level of adjustments** | **Odds ratio (95% CI)** | **p** |
| --- | --- | --- |
| No adjustments | 7.25 (1.90-27.64) | 0.004 |
| Age | 7.43 (1.92-28.76) | 0.004 |
| Age, sex | 10.61 (2.31-48.66) | 0.002 |
| Age, sex, complete heart block, atrial fibrillation, hypertension, ischaemic heart disease, diabetes | 14.62 (2.09-102.32) | 0.007 |
| Age, sex, complete heart block, atrial fibrillation, hypertension, ischaemic heart disease, diabetes, QRS duration | 14.59 (1.69-125.62) | 0.015 |
| Age, sex, complete heart block, atrial fibrillation, hypertension, ischaemic heart disease, diabetes, QRS duration, NYHA class | 24.77 (1.78-344.70) | 0.017 |
| Age, sex, complete heart block, atrial fibrillation, hypertension, ischaemic heart disease, diabetes, QRS duration, NYHA class, Cum%VP | 19.09 (1.37-266.34) | 0.028 |
| **Supplementary table.** Effect of global longitudinal strain <14.5 compared to >14.5 on risk of decline in left ventricular ejection fraction >5% at 12 months according to level of adjustments. | | |
